# Feeling fooled: Texture contaminates the neural code for tactile speed

**DOI:** 10.1371/journal.pbio.3000431

**Published:** 2019-08-27

**Authors:** Benoit P. Delhaye, Molly K. O'Donnell, Justin D. Lieber, Kristine R. McLellan, Sliman J. Bensmaia

**Affiliations:** 1 Department of Organismal Biology and Anatomy, University of Chicago, Chicago, Illinois, United States of America; 2 Institute of Neuroscience, Université catholique de Louvain, Brussels, Belgium; 3 Committee on Computational Neuroscience, University of Chicago, Illinois, United States of America; Yeshiva University Albert Einstein College of Medicine, UNITED STATES

## Abstract

Motion is an essential component of everyday tactile experience: most manual interactions involve relative movement between the skin and objects. Much of the research on the neural basis of tactile motion perception has focused on how direction is encoded, but less is known about how speed is. Perceived speed has been shown to be dependent on surface texture, but previous studies used only coarse textures, which span a restricted range of tangible spatial scales and provide a limited window into tactile coding. To fill this gap, we measured the ability of human observers to report the speed of natural textures—which span the range of tactile experience and engage all the known mechanisms of texture coding—scanned across the skin. In parallel experiments, we recorded the responses of single units in the nerve and in the somatosensory cortex of primates to the same textures scanned at different speeds. We found that the perception of speed is heavily influenced by texture: some textures are systematically perceived as moving faster than are others, and some textures provide a more informative signal about speed than do others. Similarly, the responses of neurons in the nerve and in cortex are strongly dependent on texture. In the nerve, although all fibers exhibit speed-dependent responses, the responses of Pacinian corpuscle–associated (PC) fibers are most strongly modulated by speed and can best account for human judgments. In cortex, approximately half of the neurons exhibit speed-dependent responses, and this subpopulation receives strong input from PC fibers. However, speed judgments seem to reflect an integration of speed-dependent and speed-independent responses such that the latter help to partially compensate for the strong texture dependence of the former.

## Abstract 2

Our ability to sense the speed at which a surface moves across our skin is highly unreliable and depends on the texture of the surface. This study shows that speed illusions can be predicted from the responses of a specific population of nerve fibers and of their downstream targets; because the skin is too sparsely innervated to compute tactile speed accurately, the nervous system relies on a heuristic to estimate it.

## Introduction

When we manipulate objects, we acquire information about their properties—their shape and texture, e.g.—through the sense of touch. Most interactions involve movement between skin and object, and tactile signals tend to be enhanced by movement, leading, for example, to increased sensitivity to surface texture [1–3] and to local object contours [4]. We are implicitly aware of the motion-induced sensitization of touch, as evidenced by the fact that we spontaneously deploy exploratory procedures that involve motion to acquire object-specific information [5]. Touch also conveys information about the motion itself, as evidenced by the fact that (blindfolded) subjects can distinguish the direction [6–8] and speed at which objects move across the skin [9,10].

Very little is known about how motion is represented in the responses of tactile nerve fibers. One conjecture is that motion is computed from the sequential activation of fibers with spatially displaced receptive fields (RFs) [11], but this has not been systematically investigated. The firing rate of nerve fibers tends to increase as stimuli move faster across the skin [12–14], but surface texture also modulates afferent firing rates. How speed- and texture-dependent components are disentangled remains to be elucidated.

In somatosensory cortex, the neural representation of motion direction has been shown to be remarkably similar to its visual counterparts [15–18]. Indeed, one subpopulation of somatosensory neurons is tuned for the direction of motion of individual stimulus contours—individual stimulus edges, e.g.—whereas a (presumably downstream) population is tuned for the direction of the stimulus as a whole, a motif that is also observed in primary visual and medial temporal cortices [17,19]. In fact, the same computation seems to be deployed in the two sensory systems to compute global motion direction from local motion signals.

Less is known, however, about the neural basis of tactile speed coding. One of the key findings from extant studies, however, is that, although the responses of cortical neurons are modulated by scanning speed, their responses also tend to be dependent on surface texture, similar to their afferent counterparts. Although the texture dependence of cortical responses may underlie the texture dependence of tactile speed perception, this relationship is not straightforward [20]. Furthermore, previous studies of speed coding used coarse textures, which provide an incomplete picture of tactile coding because they exclude neural mechanisms involved in processing fine surface elements (smaller than approximately 0.5 mm), thereby disregarding most of the tangible range of spatial scales [1,21].

The objective of the present study is to elucidate the neural mechanisms of speed coding along the somatosensory neuraxis using the full range of tangible textures. First, we measure the ability of human subjects to judge and discriminate tactile speed to characterize the perceptual sensitivity to speed and its dependence on surface texture. Second, we examine the responses of tactile nerve fibers to scanned surfaces, characterize how scanning speed modulates the response, and ascertain what aspects of the response might account for speed perception. Finally, we examine the speed and texture dependence of the responses of neurons in somatosensory cortex, investigate how these responses might be inherited from their peripheral inputs, and assess how the cortical representation might account for perception. We conclude that tactile speed is not explicitly computed from the responses of tactile nerve fibers, contrary to existing models of speed processing. Rather, perceived speed is determined by the relative strength of afferent responses, themselves systematically dependent on surface texture. Our tactile perception of speed is thus predictably nonveridical.

## Results

To elucidate the neural mechanisms of tactile speed processing, we obtained from blindfolded human subjects perceptual judgments of speed as a variety of textured surfaces (see S1 Table) were scanned across their stationary fingertips using a circular drum. Scanning speeds ranged from 40 mm/s to 120 mm/s, spanning the range of speeds used spontaneously [22–24]. In parallel experiments, we recorded the responses of tactile nerve fibers and of neurons in the somatosensory cortex of primates to an overlapping set of textures using an identical apparatus. We then assessed how speed affected the neuronal responses in the nerve and in the brain, the degree to which speed-related responses could be disentangled from texture-related ones, and the extent to which neuronal responses could account for perceptual judgments of speed. Note that neuronal responses from rhesus macaques have been previously shown to yield very precise predictions of the perception of motion direction in humans [17,19,25]. These previous results, and many others linking human perception to monkey neurophysiology, demonstrate that motion processing in macaques is a good model of its counterpart in humans.

### Psychophysics

First, we sought to assess the precision with which human subjects can judge tactile speed and the degree to which these judgments depend on surface texture.

**Fig 1:**
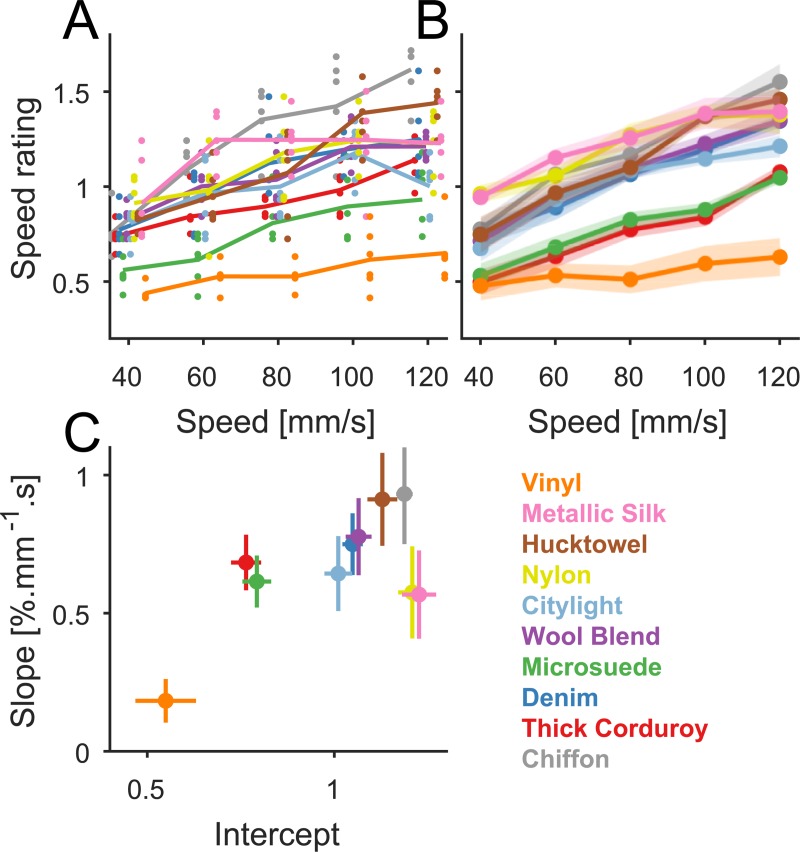
Speed ratings across textures. (A) Magnitude estimates of speed obtained from an example subject. Normalized speed ratings for different textures are shown in different colors. Dots denote single trials (*n* = 6 per condition), and lines denote means. (B) Average speed rating for all subjects (*n* = 10). Dots denote the mean, and shaded areas denote the SEM. (C) Coefficients (slope versus intercept) of the linear regression relating speed ratings to speed. Error bars denote the SEM (*n* = 10). Data underlying this figure can be found in S1 Data.

In the first experiment, textures were scanned across the subjects’ fingertip at each of five equally spaced speeds ranging from 40 to 120 mm/s, and subjects rated the speed on a scale of their own choosing. Ratings were normalized within sessions and then averaged within subjects (Fig 1A). Responses were moderately consistent across subjects (mean pairwise Pearson correlation coefficient *r* = 0.74 [*n* = 10], and *r* = 0.70 [*n* = 8] for the second subset). Critically, the perception of speed was highly influenced by texture (Fig 1B and S1A Fig): although all surfaces were perceived as moving faster as speed increased, some surfaces were systematically perceived as moving faster than others. For example, vinyl always felt slower than metallic silk. Furthermore, some textures yielded shallower speed functions than others. For example, ratings increased more slowly with speed for vinyl than for metallic silk. The texture dependence of speed ratings was quantified by examining the regression coefficients relating ratings to speed for each texture (Fig 1C and S1B Fig). Both the intercept and the slope of the regression were strongly dependent on texture. The texture-dependent effects seemed to be related to the intensity of texture-related skin vibrations, measured with a laser Doppler vibrometer, as evidenced by the correlation between vibratory power and the intercept of the regressions (S1C Fig, *r* = 0.65, *p* = 0.041, *n* = 10). This relationship between speed and skin vibrations is consistent with a previous psychophysical observation that imposing vibrations on the skin impairs tactile speed discrimination [26].

**Fig 2:**
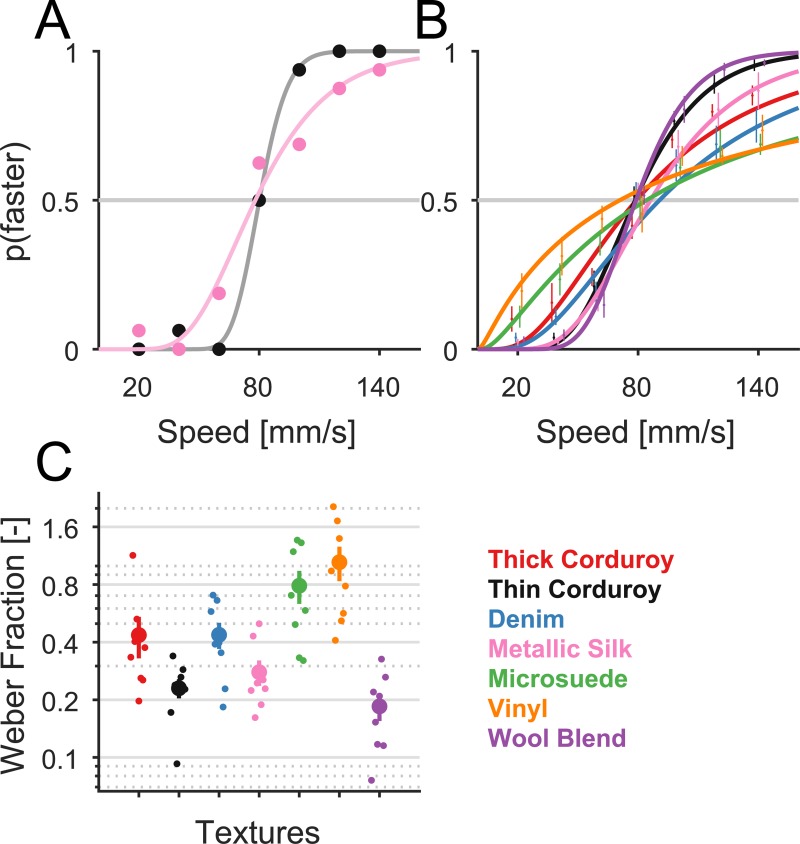
Speed discrimination within texture. (A) Discrimination performance—as gauged by the proportion of trials judged faster than the reference (at 80 mm/s) versus speed—of an example subject for “thin corduroy” (black) and “metallic silk” (pink). (B) Performance averaged across subjects for all tested textures (*n* = 8 subjects for each texture). Error bars denote the SEM. (C) Weber fraction obtained from individual subjects (small dots) along with the respective means ± SEM (large dots and bars). Data underlying this figure can be found in S1 Data.

Next, to obtain a more objective measure of the perceptual sensitivity to speed and its dependence on surface texture, we had subjects perform a discrimination task, in which they were presented with pairs of scanned surfaces—a reference at 80 mm/s and a comparison whose speed varied from 20 to 140 mm/s—and judged which surface moved faster. In some experimental blocks, the same texture was presented twice on each trial; in other experimental blocks, two different textures were presented. For each subject and texture, we fit a psychometric function to the proportion of times the comparison surface was judged to be faster than the reference as a function of speed (**Fig 2A**). Consistent with results from the magnitude estimation experiment, we found that different textures yielded very different psychometric functions (**Fig 2B**). Weber fractions—the proportion by which speed needed change for the subject to reliably judge relative speed—varied over a fivefold range (from 0.2 to 1, **Fig 2C**). As expected, Weber fractions were correlated with the slopes obtained in the magnitude estimation experiment (**S2A Fig**, *r* = −0.81, *p* = 0.052, *n* = 10).

**Fig 3:**
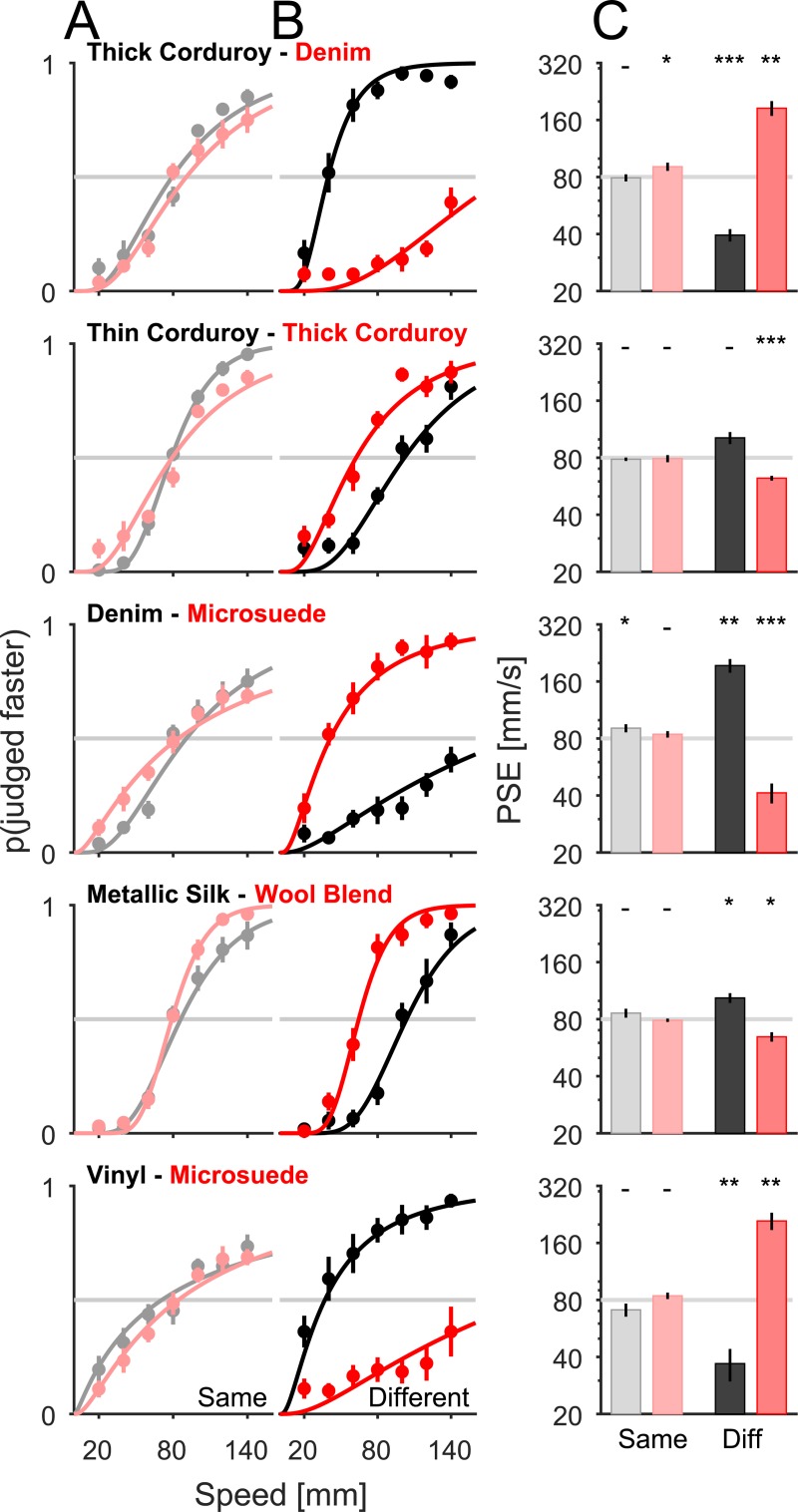
Speed discrimination across textures. (A, B) Discrimination performance—as gauged by the proportion of trials on which the comparison was judged to be faster than the reference versus speed—averaged across subjects for each texture pair. In (A), the reference and comparison were the same texture (reproduced from **Fig 2**, but organized by pairs). In (B), the reference and comparison were different textures. The color (black or red) depicts the texture used as reference. (C) PSE for the same (left, light) and different (right, dark) texture pairs. Error bars denote the SEM (*n* = 8 for each same pair, *n* = 9 subjects for each different pair). Stars reflect the outcome of a one-sample *t* test versus 80 mm/s (− denotes not significant, *p < 0.05, **p < 0.01, ***p < 0.001). The effect of texture on PSE was consistent with the results from the magnitude estimation experiment (Fig 1C, intercepts). Data underlying this figure can be found in S1 Data. PSE, point of subjective equality.

When the two textures in the pair were different, speed discrimination performance was strongly affected, as evidenced by a shift in the point of subjective equality (PSE) to the left or right, indicating that one of the textures was systematically judged to be faster than the other (Fig 3). For instance, denim was almost always perceived to be faster than thick corduroy; thin corduroy was judged to be slightly faster than thick corduroy, a difference likely driven by the difference in spatial frequency (cf. [10]). Whereas psychometric functions from the same-texture speed discrimination task yielded a PSE that coincided with the reference speed (7 one-sample *t* tests, *t*(7) = [−0.24; −0.89; 2.38; 1.32; 1.28; −1.62; −0.687], all *p* > 0.05 but one, *p* = 0.049, no Bonferroni correction), the PSE differed from the reference speed for speed discrimination with the two different textures (10 one-sample *t* tests, *t*(8) = [−13.61; 2.95; 7.11; 3.86; −5.99; 6.31; −9.85; −7.76; −4.19; 5.91], all *p* < 0.05 but one, *p* = 0.21, after Bonferroni correction). There was no relationship between Weber fractions derived from the “same-texture” trials (Fig 2C) and the shift in PSE observed in the “different-texture” trials (S2B Fig, *r* = 0.19, *p* = 0.25, *n* = 45), suggesting that speed sensitivity does not predict perceptual biases.

In summary, then, some textures provide more information about speed than do others—as evidenced by a wide range of texture-specific Weber fractions—and some textures are felt as moving faster than are others—as evidenced by shifts in the PSE when different textures are paired.

### Speed coding in the nerve

**Fig 4:**
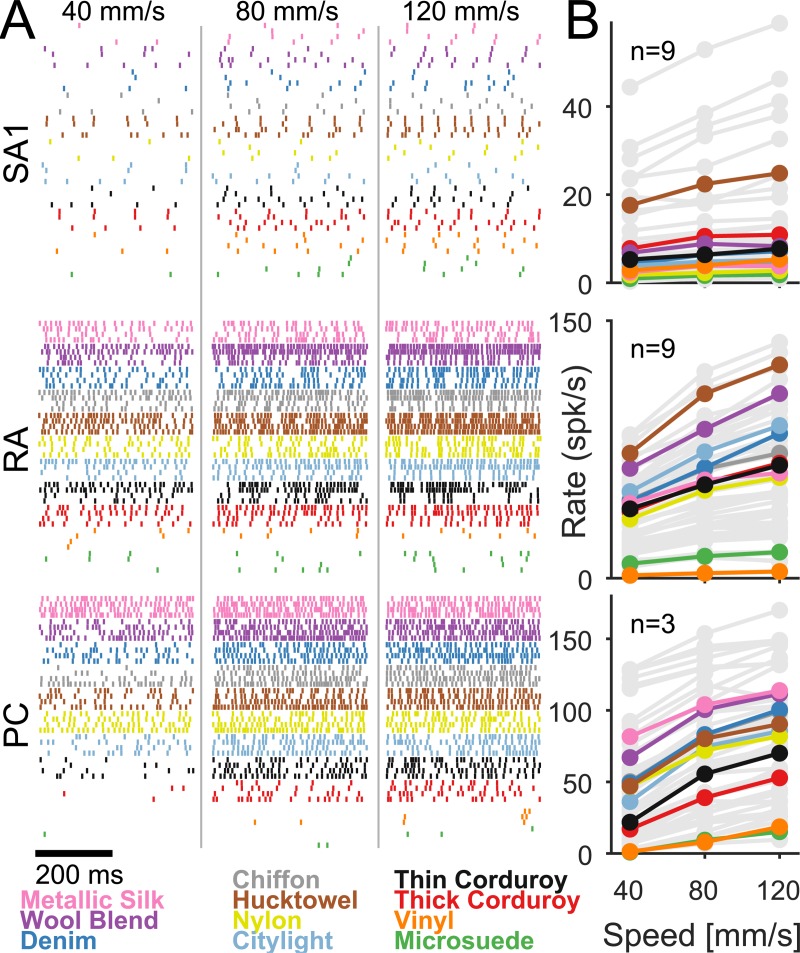
Speed dependence of the responses of tactile nerve fibers. (A) Spiking responses of two nerve fibers from each class (SA1, RA, and PC fibers) to two repeated presentations of 11 different textures scanned at three different speeds. (B) Mean firing rate across fibers of each type. Each color is a different texture (see legend at the bottom left); light gray represents the textures not used in the psychophysical experiments. Data underlying this figure can be found in S1 Data. PC, Pacinian corpuscle–associated; RA, rapidly adapting; SA1, slowly adapting type 1.

Next, we investigated the effect of changing scanning speed on the responses of tactile nerve fibers. To this end, we recorded the activity of 21 nerve fibers—9 slowly adapting type 1 (SA1), 9 rapidly adapting (RA), and 3 Pacinian corpuscle–associated (PC) fibers—as textures were scanned at different speeds across the fingertips of anesthetized monkeys. We observed that the firing rate of all afferent types increased with speed, but to different degrees (Fig 4): PC fiber responses were far more dependent on speed than were their SA1 counterparts, as evidenced by shallow functions relating mean firing rate to speed for SA1 fibers and steep ones for PC fibers (S3A Fig); RA fiber responses exhibited an intermediate degree of speed dependence. Importantly, the dependence of afferent firing rates on speed was dwarfed by their dependence on texture (Fig 4B). Indeed, mean afferent firing rate was a poor predictor of scanning speed (linear regression, *R*^*2*^ = 0.03, 0.13, and 0.19 for SA1, RA, and PC fibers, respectively).

**Fig 5:**
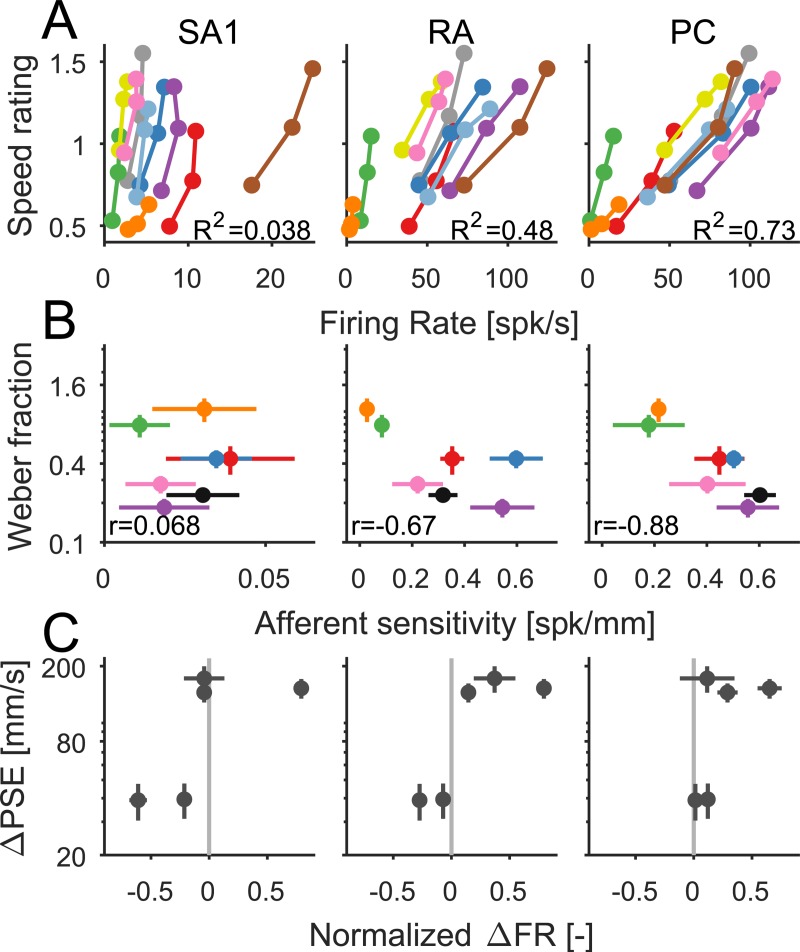
Speed coding in the nerve. (A) Rating of perceived speed (averaged across subjects) as a function of afferent FR (averaged across nerve fibers of each type) at the three tested speeds. Each color denotes a different texture (see legend in Fig 4). (B) Weber fractions as a function of afferent speed sensitivity (as indexed by the slope of the function relating afferent FR to speed). (C) Difference in PSE as a function of difference in FR at 80 mm/s (normalized using [a − b] / [a + b]). For all three psychophysical paradigms—speed rating, within-texture discrimination, and across-texture discrimination—PC FRs best account for the texture dependence of speed perception. Error bars denote the SEM. Data underlying this figure can be found in S1 Data. FR, firing rate; PC, Pacinian corpuscle–associated; PSE, point of subjective equality; RA, rapidly adapting; SA1, slowly adapting type 1; spk, spike.

Next, we investigated whether afferent response could account for perceived speed across speeds and textures. We found that PC firing rates were most predictive of perceived speed (Fig 5A, linear regression *R*^*2*^ = 0.73, *F*[1,28] = 74.51, *p* < 0.001), RA responses less so (*R*^*2*^ = 0.48, *F*[1,28] = 25.82, *p* < 0.001), and SA1 responses not at all (*R*^*2*^ = 0.02, *F*[1,28] = 0.71, *p* = 0.41). Predictions based on the responses of all three afferent types were no better than those based on PC responses alone (S3B Fig, *R*^*2*^ = 0.73; also see S3C–S3E Fig). Furthermore, PC responses could account for differences in the texture-dependent perceptual sensitivity to speed. Indeed, Weber fractions were lower when changes in speed had a strong effect on the firing rate for PC fibers (*R*^*2*^ = 0.79) and less so for RA fibers (*R*^*2*^ = 0.45) (Fig 5B). Finally, the shift in the PSE observed when subjects discriminated the speed of different textures was consistent with the relative PC fiber response to the two textures, at least in their sign (Fig 5C). That is, the texture that evoked a stronger response in PC fibers was systematically perceived as moving faster than the other.

Finally, we examined whether precise spike timing conveys information about speed. To this end, we used a cross-validated multiple regression analysis to decode the scanning speed from spiking responses of tactile fibers by comparing spike trains at different temporal resolutions using a spike distance metric (see Materials and methods). Such a metric will yield a shorter distance to the extent that two spike trains exhibit the same temporal pattern and might therefore improve performance over a simple rate code. We found that our ability to decode speed from afferent responses did not improve when timing was taken into consideration (S4A Fig) and that the speed decoded using spike timing–sensitive decoders was not more predictive of perceived speed than was that based on spike counts (S4B Fig). Temporal spiking patterns have been shown to be highly texture dependent [21], so it is not surprising that spike timing does not contribute reliable information about speed.

In summary, the firing rates of tactile nerve fibers systematically increase with increasing speed, but the perceptual sensitivity to speed and the dependence of perceived speed on surface texture seem to reflect PC firing rates.

### Speed coding in cortex

Finally, we investigated the effect of changing scanning speed on the responses of neurons in somatosensory cortex. To this end, we recorded from 49 neurons in the somatosensory cortex of awake rhesus macaques (14 in area 3b, 26 in area 1, and 9 in area 2) as we scanned 10 textures—a subset of those used in the psychophysical and peripheral nerve experiments—across their fingertips at 4 different speeds (60, 80, 100, and 120 mm/s).

#### Single neurons

**Fig 6:**
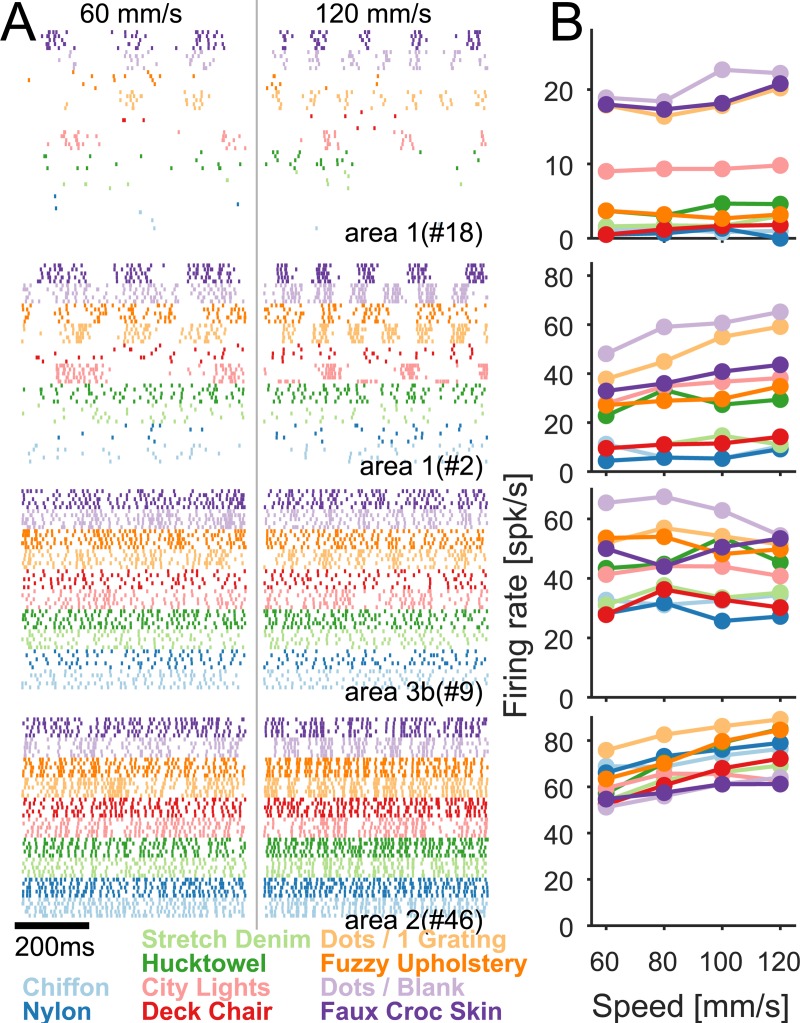
Speed dependence of the responses of cortical neurons. (A) Spiking responses of four neurons in somatosensory cortex to five repeated presentations of 10 different textures scanned over the center of their RFs at two different speeds. Neurons #2 and #46 increase their rate with speed; neurons #18 and #9 do not. (B) Mean firing rate versus speed. Each color denotes a different texture (see legend at the bottom left). Data underlying this figure can be found in S1 Data. spk, spike.

**Fig 7:**
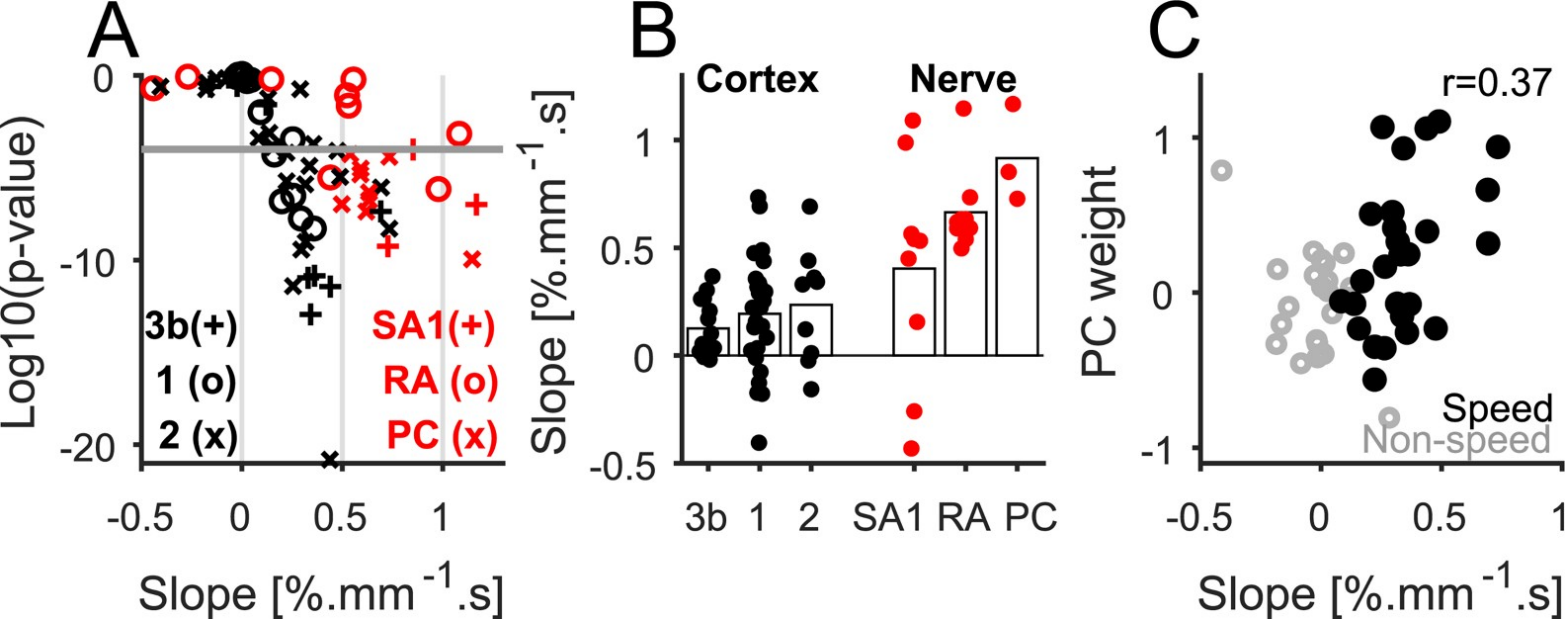
Speed coding in single cortical units. (A) *p*-Value versus normalized regression slopes for each neuron. Black denotes cortical neurons, and red denotes nerve fibers. Marker type denotes cortical field or fiber type (see legend). The gray line denotes the threshold for significance (10^−4^). (B) Regression slope for each neuron, classified by area and fiber type (bars show the mean across neurons). (C) PC weight as a function of regression slope for each neuron. Neurons with higher PC input tend to be more sensitive to speed. Data underlying this figure can be found in S1 Data. PC, Pacinian corpuscle–associated; RA, rapidly adapting; SA1, slowly adapting type 1.

First, we investigated the degree to which the responses of individual cortical neurons are modulated by scanning speed. We found that, although neurons are almost always sensitive to texture [27], speed sensitivity is much less prevalent in somatosensory cortex (Fig 6). Indeed, the responses of most neurons were significantly modulated by texture (two-way ANOVA on each neuron, *p* < 0.001 for 48/49 neurons), but the responses of only 60% of them were significantly modulated by speed (*p* < 0.001 for 29/49 neurons, see examples in Fig 6A and 6B). The majority of speed-sensitive neurons exhibited an increase (and none a significant decrease) in firing rate with speed (Fig 7A, *p* < 0.001 for 25/29 neurons). No neurons exhibited speed tuning, as evidenced by the fact that a quadratic function did not provide a better description of the relationship between firing rate and speed than did a linear one (S5A Fig). Speed sensitivity was approximately equivalent across the different cortical fields in somatosensory cortex and lower than in the nerve (Fig 7B). As expected, neurons that received strong PC fiber input tended to be more speed sensitive than neurons that did not (Fig 7C, see Materials and methods).

#### Speed signals in cortical populations

**Fig 8:**
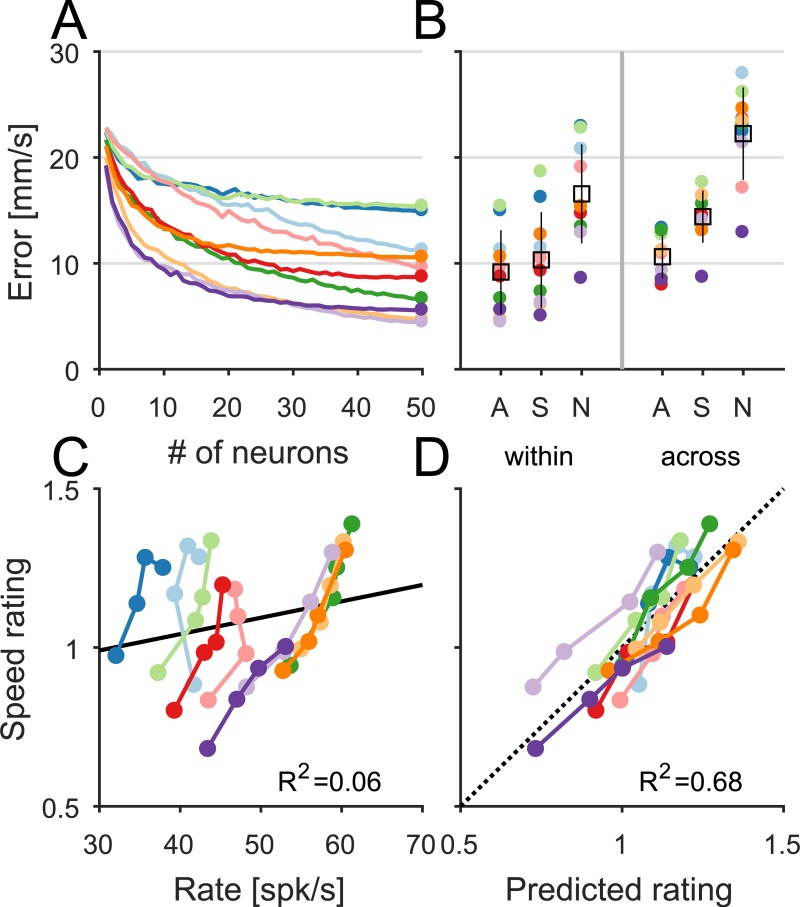
Population coding of speed in somatosensory cortex. (A) Speed decoding performance as a function of number of neurons within texture, including both speed-sensitive and speed-insensitive neurons. Each color denotes a different texture (see legend in Fig 6). (B) Speed decoding performance within (left) and across (right) textures, using A (*n* = 49), S (*n* = 25), and N (*n* = 24). Each colored dot is a different texture; the square and line denote the mean ± SD. (C) Speed ratings versus the mean firing rate of the entire cortical population. Each color denotes a different texture. (D) Speed rating versus the best-fitting linear combination of the mean firing rates of the two subpopulations of neurons (speed sensitive and insensitive). Data underlying this figure can be found in S1 Data. A, the entire population of neurons; N, the speed-insensitive subpopulation; S, the speed-sensitive subpopulation; spk, spike.

Next, we sought to estimate the degree to which speed can be veridically decoded from the firing rates of populations of cortical neurons. First, we found that the ability to decode speed from neuronal responses leveled off with as few as 20 neurons (Fig 8A, fitted half-life mean ± SD: 9.3 ± 5.1 neurons within texture and 14.7 ± 11.9 neurons across texture), but the asymptotic performance level varied from texture to texture. As expected, within-texture decoding performance was driven by the speed-sensitive neurons (Fig 8B, left): performance using only this population (*n* = 25) was similar to the performance using the entire population (*n* = 49). However, when decoding speed across textures, the inclusion of the speed-insensitive population significantly boosted performance (Fig 8B, right), suggesting that the texture confound can be corrected for using the responses of neurons that encode texture but not speed. With speed-insensitive responses included, performance across texture was similar to that within texture.

#### The neural basis of speed perception in somatosensory cortex

Given that speed perception is strongly texture dependent, we did not expect to find a veridical speed signal in somatosensory cortex. Rather, we wished to find an aspect of the cortical response that could account for the speed judgments obtained from human subjects. First, we examined whether the population firing rate—which is sensitive to both speed and texture—might account for the speed perception and found that it did not (Fig 8C, *R*^*2*^ = 0.06, *F*[1,38] = 2.43, *p* = 0.13), nor could it account for the texture-dependent bias in speed discrimination (S5B Fig, *r* = −0.07, *p* = 0.85, *n* = 10). Then, we examined whether speed perception might be exclusively determined by the responses of speed-sensitive neurons but found that the response of this population overestimated the texture dependence of speed ratings. In light of this, we examined whether the combined responses of speed-sensitive and speed-insensitive neurons might drive perceived speed. Specifically, we assessed whether a linear combination of the mean firing rates in the two populations might predict perceived speed and found that it did (Fig 8D, *R*^*2*^ = 0.68, *F*[2,37] = 39.59, *p* < 0.001). The speed neurons’ weight (0.030) and the non–speed neurons’ weight (−0.027) were similar in amplitude but of opposite sign, suggesting that the non–speed neurons partially compensate for the bias introduced by the different textures. In other words, the texture modulation in the responses of speed-sensitive neurons is compensated for to some degree based on the responses of speed-insensitive neurons.

## Discussion

We show that the speed perception depends on texture in two ways. First, some textures systematically feel as though they are moving faster than others. This strong dependence of speed perception on surface texture is surprising given the near independence of texture perception on speed [28]. Second, some textures provide more accurate speed signals. We find that PC responses account for both of these aspects of the texture dependence of speed perception, suggesting that these fibers play a critical role in speed processing. In somatosensory cortex, neurons can be split into two populations: neurons whose responses increase monotonically with scanning speed—which receive dominant input from PC fibers (Fig 7C)—and neurons whose responses are insensitive to speed. Speed perception depends on both populations, in which the speed-independent signal serves to partially correct for the texture dependence of the speed-dependent signal.

### An intensive code for tactile speed

Previous work has shown that the coding of motion direction is similar in visual and somatosensory cortices [16,29]. At early stages of motion processing, cortical neurons are tuned for the direction of local edges. However, these signals are subject to the aperture problem and convey ambiguous information about the direction of global motion. In both sensory modalities, the ambiguity seems to be resolved in similar ways, suggesting that similar computations are implemented to solve the same problem [15].

The perception of motion speed also bears some similarities in the two modalities. For example, high-contrast visual gratings are perceived to move faster than low-contrast ones [30], an effect that is analogous to the effect of vibratory intensity on perceived tactile speed. Furthermore, the spatial frequency of visual gratings influences their perceived speed [31–33], a phenomenon that is also observed in touch [10]. In fact, frequency provides a common frame of reference to integrate visual and tactile speed signals [34].

However, the processing of motion speed seems to rely on fundamentally different mechanisms in the visual and somatosensory systems. Indeed, neurons in primary visual cortex exhibit tuning to speed [35,36], and this tuning persists through multiple stages of processing along the visual pathway [37–39]. Speed tuning is thought to stem from elementary motion detectors (EMDs), consisting of two receptors with spatially displaced RFs whose outputs are temporally delayed relative to each other and combined multiplicatively [40–42]. The preferred speed—which elicits the highest firing rate—is not only determined by the spacing between RFs and the time delay but also influenced by the contrast and spatial frequency of the object [39,43]. The similarities between direction coding and perception in vision and touch [7,11,17,44,45] suggest the presence of an EMD-like mechanism to compute motion direction.

Tactile speed, on the other hand, is not computed by EMDs, as evidenced by the lack of speed tuning in cortex [19,20]. Computational modeling reveals that an EMD-like mechanism appears ill-suited to compute tactile speed given the range of speeds experienced during everyday manual interactions. Indeed, the density of innervation of the skin is too sparse and the required delays too long for such a mechanism to mediate the extraction of tactile speed (S6 Fig). Rather, we propose that the perceived speed of a surface is determined by the strength of the neural response, both in the nerve—primarily by PC fiber afferents—and in somatosensory cortex. This intensive code for tactile speed is consistent with previous psychophysical observations that motion aftereffects become stronger with increased speed [46].

Unlike an EMD-like mechanism, which actually computes speed, the intensive code for tactile speed is highly susceptible to biases because any signal that modulates PC fiber firing rates confounds it. The ambiguity of the speed representation is only partially rescued by a texture-dependent, speed-independent signal—carried by a subpopulation of neurons in somatosensory cortex—which corrects for the modulating influence of the texture-related signal on the speed-related signal. The advantage of this PC-mediated intensive code is that it is supported by a much larger population of fibers. Whereas an EMD-like mechanism would rely on 100 or 200 nerve fibers with RFs over the area of contact with the surface, skin vibrations elicited during texture scanning propagate across the skin over long distances, thereby exciting a lot more afferents [47,48].

### Temporal code for tactile speed

We tested two putative spike timing–dependent codes for tactile speed: the EMD-like mechanism and a more generic readout based on temporal spiking sequences. The first was rejected because its implementation is biologically implausible (S6 Fig). The second was rejected because it did not yield improved speed decoding or better predictions of perceived speed (S4 Fig). That speed perception is not mediated by a temporal code is not surprising given the critical dependence of spike timing on texture [21]. Indeed, different textures evoke different temporal spiking patterns, which dilate or contract with decreases or increases in scanning speed, respectively. Speed information can only be extracted from these spiking sequences if the contribution of the texture to that sequence is known. We did not test every possible approach to extract speed information from temporal spiking patterns, so there may be a way to do so. However, the present results suggest that a simple code based on firing rates accounts for the dependence of perceived speed on speed and texture.

### Conclusions

The main conclusion of this study, then, is that speed coding relies on a heuristic: the stronger the skin vibrations, the faster a surface is perceived as moving across the skin. Although an EMD-like mechanism provides an objective computation of speed, the peripheral apparatus is ill-suited for this computation given the range of speeds experienced during manual interactions. Rather, the strength of the response in PC fibers and in their downstream targets determines how fast a surface is perceived as moving across the skin. Because it relies on a heuristic, the speed signal is highly unreliable and depends strongly on other factors, including surface texture. During manual interactions with objects, this cutaneous signal about speed is complemented by a sensorimotor signal about the exploratory movements. These two signals together may yield more veridical information about the relative motion between skin and surface.

## Materials and methods

### Ethics statement

All human procedures were approved by the Institutional Review Board (IRB15-1670), and all subjects (50 subjects in total) provided signed informed consent.

All animal procedures were approved by the Institutional Animal Care and Use Committee of the University of Chicago (ACUP #72042).

### Stimuli

Textured surfaces were diverse, ranging from fine to coarse, including fabrics, embossed dots, and gratings (see S1 Table) [21,49,50]. Each textured strip (2.5 cm wide × 16 cm long) was wrapped around an acrylic drum (25.4 cm in diameter and 30.5 cm in length) to cover its surface completely. The drum was mounted on a combination of a horizontal stage (PRO115-05MM-400, Aerotech, Pittsburgh, PA, USA), a vertical stage (PRO115-05MM-150, Aerotech), and a rotating motor (SM2316DT-PLS2 SmartMotor, Moog Animatics, Santa Clara, CA, USA) to allow for the automatic presentation of any texture strip at a prespecified contact force and scanning speed. On each trial, the drum was first accelerated to its desired rotational speed and then lowered onto the fingertip at a vertical position that was precalibrated for each texture to exert a contact force of 0.25 N (±0.1 N). For all psychophysical experiments described below, the duration of each stimulus was the same across speeds textures (0.8 s) such that the subjects could not infer speed from the duration of the stimulus.

### Psychophysics

#### Procedures

For all experiments, subjects sat in front of the drum with their right arm supinated and resting on a support such that the hand was placed under the drum. The index finger was strapped on a support such that the drum precisely made contact with the same portion of the fingertip and with the prespecified amount of force on each trial. A curtain prevented the view of the textures and the drum. White noise was played through computer speakers, and subjects wore sound-blocking headphones to mask sounds from the motors or from the skin’s interaction with the surface [51].

#### Speed estimation

On each trial, subjects (*n* = 10, 8 females, 24.6 y old on average) were passively presented with one of 10 surfaces (thick corduroy, stretch denim, microsuede, wool blend, city lights, nylon, huck towel, metallic silk, vinyl, and chiffon) and provided a rating proportional to its perceived speed. If a surface was perceived to move twice as fast as another, they were to ascribe to it a rating that was twice as high. Subjects could use a numerical scale of their choosing and were encouraged to use decimals or fractions. They were explicitly instructed to ignore the texture and to focus on the perceived speed. Each texture was presented twice per experimental block at six different speeds (40, 60, 80, 100, or 120 mm/s). The texture and speed order were randomly permuted. Each subject participated in three experimental blocks, leading to six repeats of each texture and each speed (5 speeds × 10 textures × 6 repetitions = 300 trials). Ratings were normalized by their within-block mean and then averaged across repetitions to yield one value per texture, speed, and subject.

The same experiment was repeated (*n* = 9, 5 females, 22.4 years old on average) with a different subset of 10 textures that exactly matched those used in the cortical experiment (chiffon, nylon, stretch denim, huck towel, city lights, deck chair, mixed dots/grating, fuzzy upholstery, dots, faux croc skin). Five textures were common to both sets. Data from one subject were discarded because he did not understand the instructions.

Given that speed ratings were linearly increasing with speed, the parameters (slope and intercept) of a linear fit were obtained for each texture and subject. For that purpose, we first subtracted 80 mm/s from the actual speeds such that the intercept reflected the rating at 80 mm/s for each texture.

#### Speed discrimination

On each trial, subjects (*n* = 31, 20 females, 22.7 years old on average) were passively presented with one pair of textures, a reference, and a comparison. The reference texture was scanned at 80 mm/s, and the comparison texture at one of seven speeds (20, 40, 60, 80, 100, 120, and 140 mm/s). The subjects judged which of the two surfaces moved faster. In a first experiment, the same texture was presented as the reference and the comparison (same texture condition). Each pair of speeds was presented 16 times, leading to 112 trials (16 repetitions × 7 speeds) per texture. In this experiment, a set of seven textures (corduroy thick ridges, corduroy thin ridges, stretch denim, metallic silk, microsuede, vinyl, wool blend) was tested in eight subjects. In a second experiment, the reference and the comparison textures were different (different-texture condition). Each pair of speeds was repeated 12 times, and each texture was systematically assigned as reference or comparison, leading to 168 trials (12 repetitions × 2 textures as reference × 7 speeds) per texture pair. Five different pairs of textures (thick corduroy–stretch denim, corduroy thin–thick ridges, stretch denim–microsuede, metallic silk–wool blend, vinyl–microsuede) were tested in nine subjects. The order of presentation of the reference and the comparison (first or second), the order of the comparison speed, and the order of texture pairs were all randomized, and all trials were typically split into three blocks with breaks in between.

The proportion of trials for which the comparison stimulus was judged to be faster than the reference was evaluated for each comparison speed. These proportions were then fit to log(speed) using a normal cumulative density function (using lsqcurvefit in Matlab). The psychometric function was composed of two free parameters: the mean (μ) and the standard deviation (σ). The two parameters were then converted to the PSE (PSE = exp[μ]) and to the Weber fraction (= σ × 0.6745, which is the relative change of speed required to reach 75% correct discrimination). The same procedure was followed for the paired experiments after splitting the data by reference texture. For the paired experiments, only the PSE was computed.

#### Vibrometry

Laser Doppler vibrometry was used to record texture-elicited vibrations on the right index finger pad of five subjects (Polytec OFV-3001 with OFV 311 sensor head, Polytec, Irvine, CA, USA). The methods have been described in a previous publication [48,49] and are only summarized here. The laser was focused on the finger pad 7–15 mm from the locus of stimulation onto a small square of white-out tape (BIC USA, Shelton, CT, USA) applied to the skin to increase signal strength by increasing reflectivity. Each of the 55 textures was scanned 10 times on the index fingertip at 80 mm/s. The data were digitized at 100 kHz, and only a window of 0.5 s made up of a steady-state skin response was analyzed. The average RMS value across subjects was obtained for each texture and averaged across repetitions after filtering the velocity signal with a second-order band-pass Butterworth filter (150 Hz–750 Hz).

### Neurophysiology

#### Peripheral recordings

Previous reports describe in detail the experimental procedures and the apparatus [21,50]. Briefly, extracellular single-unit recordings were collected from the median and ulnar nerves of six rhesus macaques using standard procedures [52,53]. Spiking responses of 17 SA1, 15 RA, and 7 PC fibers innervating the fingertips were recorded during the scanning of textures on the center of their RFs. Units were classified as SA1, RA, and PC fibers using standard methods. Textures (*n* = 55) were scanned at three different speeds (40, 80, and 120 mm/s). Each texture was presented at least twice at each speed. In total, 9 SA1, 9 RA, and 3 PC fibers were held long enough to run the entire protocol with a high signal-to-noise ratio, and only those were included for the analyses.

#### Cortical recordings

All procedures have been described in a previous publication [27] and are only summarized here. Extracellular recordings were made in the postcentral gyri of three hemispheres of three macaque monkeys using previously described techniques [54]. On each recording day, a multielectrode microdrive, loaded with three electrodes, was lowered normal to the cortical surface and driven into the cortex until they encountered neurons from Brodmann’s areas 3b, 1, and 2 of somatosensory cortex, with RFs on the distal finger pad. The different cortical fields were identified by tracking the progression of RFs across the cortical surface, as we have previously done. We recorded from neurons whose RFs were located on the distal pads of digits 2–5. On roughly every second day of recording, the electrode array was shifted 200 μm along the postcentral gyrus until the entire representation of digits 2–5 had been covered. Recordings were obtained from neurons in areas 3b, 1, and 2 that met the following criteria: (1) action potentials were well isolated from the background noise, (2) the RF of the neuron included at least one of the distal finger pads on digits 2–5, (3) the finger could be positioned such that the RF of the neuron was fully stimulated by the texture drum, and (4) the neuron was clearly driven by light cutaneous touch. A subset of 10 textures (see the Psychophysics section) was presented five times at four different speeds (60, 80, 100, and 120 mm/s).

#### Data analyses

The peripheral and the cortical data were processed in the same way. To evaluate the firing rates, we restricted the analysis to a time window that included only the steady-state response, excluding its onset and offset transients. The duration of the window depended on the stimulus speed (2, 1, and 0.5 s for 40, 80, and 120 mm/s for the peripheral responses, and 2, 1.5, 1.2, and 1 s for 60, 80, 100, and 120 mm/s for the cortical responses, respectively). Given that the firing rates increased approximately linearly with speed, the parameters (slopes and intercepts) of a linear regression of the firing rate as a function of speed were obtained for each texture and each neuron, after averaging across repetitions (as in **Fig 5**). To isolate the effect of speed from that of texture on the response (in the Speed coding in cortex section of the Results), firing rates were normalized within texture (by the grand mean of the responses to that texture across speeds and repetitions), and a regression relating normalized response—averaged across textures—to speed was computed for each neuron (as in **Fig 7**). For these regressions, speeds were first centered such that the y-intercept of the regression reflected its value at 80 mm/s.

We used cross-validated ridge regression with a leave-one-out procedure to evaluate whether taking spike timing into consideration improves our ability to decode speed from afferent responses. As we have previously done [55,56], we used a spike distance metric [57] to gauge the dissimilarity between pairs of spike trains from the same afferent over different conditions (*n* = 30, 10 textures × 3 speeds). With the cost of adding or removing a spike fixed at 1, we varied the parameter *q*—which determines the cost shifting a spike—to assess the influence of temporal precision. For each afferent, a distance matrix (30 × 30) was computed for values of *q* ranging from 1 to 1,000 s^−1^ (thus spanning temporal resolutions from 1,000 to 1 ms). We used triplets of afferents of each type, selected with replacement (*n*_rep_ = 24), to build three-element feature vectors, each obtained by summing the distance matrices (by taking the square root of the sum of squares) and then performing classical multidimensional scaling (cmdscale in Matlab) and retaining only the three first dimensions. For *q* = 0, this process is equivalent to using firing rates. For *q* > 0, the features contain information related to spike timing at a resolution determined by *q* [56]. We then used this feature vector to predict speed for the left-out condition. The ridge parameter was iteratively optimized such that it minimized the cross-validated mean squared error. The coefficient of determination (*R*^*2*^) could then be computed over all conditions, each left out in turn. We also computed the correlation between the prediction (from the afferent responses) and the average speed ratings (from the Psychophysics section) to assess the degree to which the effect of texture led to similar decoding errors.

To decode speed from the cortical population firing rates, we again used cross-validated ridge regression with the same procedures. The decoding performance was evaluated for different population sizes, starting from one neuron and increasing the population size in steps of one to the entire population (*n* = 49). At each step *k*, we randomly sampled *k* neurons from the population, fit a ridge regression model to predict the stimulus speed from the firing rates of these *k* neurons. We repeated this procedure 100 times by resampling *k* neurons randomly and reported the mean performance. We applied the same method to decode speed within and across textures.

The relative contributions of the different afferent types to the response of each neuron in somatosensory cortex were estimated with a linear model [27] using data collected at 80 mm/s. Specifically, we regressed the z-scored mean texture responses of each cortical neuron on the z-scored mean responses of SA1, RA, and PC afferents. For this analysis, we used a subset of 24 textures for which both peripheral and cortical responses had been recorded. These normalized regression weights were used as measures of the relative strength of SA1, RA, and PC afferent input into each neuron.

## Supporting information

S1 Fig Speed ratings and vibration intensity. (A, B) Speed ratings for the subset of textures used in the cortical experiments (*n* = 8). Each color denotes a different surface. (C) Intercept (averaged across subjects) versus vibratory intensity (averaged across subjects, *n* = 5). Vibration intensity is shown in dB with respect to mean intensity across textures. Textures that elicit stronger vibrations tend to be perceived as moving faster. Data underlying this figure can be found in S1 Data.
(EPS)

S2 Fig Speed ratings are related to speed discrimination performance. (A) Speed ratings and speed discrimination performance reveal similar texture dependence of speed sensitivity. Weber fractions are negatively correlated with speed regression slopes across the six shared textures. Error bars denote the SEM (*n* = 9 for Weber fractions, *n* = 10 for speed rating slopes). (B) Differences in PSE versus differences in log(Weber fraction). Differences in sensitivity, measured in the same-texture condition, do not correlate with differences in PSE, measured in the different-texture condition. Each color shows a different pair of textures. Small bright dots denote single-subject data. Large dark dots denote means. Data underlying this figure can be found in S1 Data. PSE, point of subjective equality.
(EPS)

S3 Fig Sensitivity of afferent responses to speed. (A) Slope and intercept of the regression relating the firing rate of tactile fibers to speed (SA1 fibers in green, RA fibers in blue, and PC fibers in orange). Error bars denote the SEM (*n* = 9 SA1, 9 RA, and 3 PC fibers). (B) Speed rating versus speed predicted from a multiple regression of the three afferent population average firing rates (3 predictors + 1 intercept). Each dot is the mean across all subjects. Different colors denote different textures. (C–E) Intercept of the regression relating speed rating to speed versus intercept of the regression relating afferent firing rate to speed. Perceptual biases are best predicted from PC fiber firing rates. Data underlying this figure can be found in S1 Data. PC, Pacinian corpuscle–associated; RA, rapidly adapting; SA1, slowly adapting type 1.
(EPS)

S4 Fig Contribution of precise spike timing to speed perception. (A) Cross-validated performance of a speed decoder (multiple regression) as a function of temporal resolution. Afferents’ firing rates provide poor predictions of the actual speed, and taking precise spike timing into consideration does not improve performance. Lines and shaded area denote mean ± SD across repetitions (*n* = 24 for SA1 and RA fibers, *n* = 1 for PC fibers). (B) Correlation between decoded speed and perceptual ratings. Decoding actual speed from afferent firing rates leads to errors that mirror the perceptual biases for the RA and PC afferents. Data underlying this figure can be found in S1 Data. PC, Pacinian corpuscle–associated; RA, rapidly adapting; SA1, slowly adapting type 1.
(EPS)

S5 Fig Cortical neuron responses to different speeds. (A) Cortical neurons are not tuned to speed. Difference in the AIC for a quadratic and a linear fit versus the extrema of the fitted quadratic function. Black dots denote concave downward quadratic fits (tuned responses, see inset), and red dots denote concave upward fits (untuned responses). Of the six neurons with a positive AIC difference and a “preferred speed” within the range tested, none show a peak in firing rate greater than 7% above the mean firing rate across speeds, so their “tuning” is so shallow as to be irrelevant. (B) The texture-dependent bias in the speed rating is not related to the mean population firing rate in somatosensory cortex. Perceptual bias (intercept of the regression as in S1B Fig) as a function of the normalized population firing rate for each texture. Data underlying this figure can be found in S1 Data. AIC, Akaike information criterion.
(EPS)

S6 Fig An EMD-like mechanism cannot work for tactile speed. (A) The (x, y) coordinates of 240 afferents were obtained after uniformly sampling within a patch of skin with a 1-cm^2^ circle. In total, 240 units per cm^2^ is the maximum afferent density at the fingertips [58]. The distance between all possible pairs of afferents was computed and is shown as a histogram. (B) Typical exploration velocity statistics, obtained from a lognormal distribution centered on 30 mm/s [24]. (C) Statistics of the resulting integration time (ratio of distance to velocity). The median time is approximately 150 ms, which far exceeds plausible times for a coincidence detector [59]. Data underlying this figure can be found in S1 Data. EMD, elementary motion detector.
(EPS)

S1 Data Data associated with Figs 1–8 and S1–S6 Figs. (XLSX)

S1 Table List of textures and experiments. (DOCX)

## Abbreviations

EMD: elementary motion detector
PC: Pacinian corpuscle–associated
PSE: point of subjective equality
RA: rapidly adapting
RF: receptive field
SA1: slowly adapting type 1

## References

[1] Hollins M, Risner SR. Evidence for the duplex theory of tactile texture perception. Percept Psychophys. 2000;62: 695–705. 10.3758/BF03206916 10883578

[2] Bensmaia SJ, Hollins M. The vibrations of texture. Somatosens Mot Res. 2003;20: 33–43. 10.1080/0899022031000083825 12745443 PMC2074877

[3] Lederman SJ. Tactual roughness perception: Spatial and temporal determinants. Can J Psychol Can Psychol. 1983;37: 498–511. 10.1037/h0080750

[4] Craig JC. Identification of scanned and static tactile patterns. 2002;64: 107–120. 10.3758/bf03194560 11916294

[5] Klatzky RL, Lederman SJ, Hamilton C, Grindley M, Swendsen RH. Feeling textures through a probe: effects of probe and surface geometry and exploratory factors. Percept Psychophys. 2003;65: 613–31. 12812283 10.3758/bf03194587

[6] Essick GK, Bredehoeft KR, McLaughlin DF, Szaniszlo JA. Directional sensitivity along the upper limb in humans. Somatosens Mot Res. 1991;8: 13–22. 10.3109/08990229109144725 2048360

[7] Essick GK, Whitsel BL. Factors influencing cutaneous directional sensitivity: A correlative psychophysical and neurophysiological investigation. Brain Res Rev. 1985;10: 213–230. 10.1016/0165-0173(85)90025-6 3938308

[8] Gardner EP, Sklar BF. Discrimination of the direction of motion on the human hand: a psychophysical study of stimulation parameters. J Neurophysiol. 1994;71: 2414–29. 10.1152/jn.1994.71.6.2414 7931525

[9] Essick GK, Franzen O, Whitsel BL, Franzén O, Whitsel BL, Franzen O, et al Discrimination and scaling of velocity of stimulus motion across the skin. Somatosens Mot Res. 1988;6: 21–40. 10.3109/08990228809144639 3242342

[10] Dépeault A, Meftah E-M, Chapman CE. Tactile speed scaling: contributions of time and space. J Neurophysiol. 2008;99: 1422–34. 10.1152/jn.01209.2007 18199814

[11] Gardner EP, Costanzo RM. Neuronal mechanisms underlying direction sensitivity of somatosensory cortical neurons in awake monkeys. J Neurophysiol. 1980;43: 1342–54. 10.1152/jn.1980.43.5.1342 6768850

[12] Essick GK, Edin BB. Receptor encoding of moving tactile stimuli in humans. II. The mean response of individual low-threshold mechanoreceptors to motion across the receptive field. J Neurosci. 1995;15: 848–864. 7823185 10.1523/JNEUROSCI.15-01-00848.1995 PMC6578277

[13] Edin BB, Essick GK, Trulsson M, Olsson KA. Receptor encoding of moving tactile stimuli in humans. I. Temporal pattern of discharge of individual low-threshold mechanoreceptors. J Neurosci. 1995;15: 830–847. 7823184 10.1523/JNEUROSCI.15-01-00830.1995 PMC6578302

[14] Greenspan JD. Influence of velocity and direction of surface-parallel cutaneous stimuli on responses of mechanoreceptors in feline hairy skin. J Neurophysiol. 1992;68: 876–889. 10.1152/jn.1992.68.3.876 1432054

[15] Pack CC, Bensmaia SJ. Seeing and Feeling Motion: Canonical Computations in Vision and Touch. PLoS Biol. 2015;13: e1002271 10.1371/journal.pbio.1002271 26418156 PMC4587910

[16] Pei Y-C, Hsiao SS, Bensmaia SJ. The tactile integration of local motion cues is analogous to its visual counterpart. Proc Natl Acad Sci U S A. 2008;105: 8130–5. 10.1073/pnas.0800028105 18524953 PMC2430371

[17] Pei Y-C, Hsiao SSS, Craig JC, Bensmaia SJ. Shape invariant coding of motion direction in somatosensory cortex. PLoS Biol. 2010;8: e1000305 10.1371/journal.pbio.1000305 20126380 PMC2814823

[18] Bicchi A, Scilingo EP, Ricciardi E, Pietrini P. Tactile flow explains haptic counterparts of common visual illusions. Brain Res Bull. 2008;75: 737–741. 10.1016/j.brainresbull.2008.01.011 18394519

[19] Pei Y-C, Hsiao SSS, Craig JC, Bensmaia SJ. Neural mechanisms of tactile motion integration in somatosensory cortex. Neuron. 2011;69: 536–547. 10.1016/j.neuron.2010.12.033 21315263 PMC3052381

[20] Dépeault A, Meftah E-M, Chapman CE. Neuronal correlates of tactile speed in primary somatosensory cortex. J Neurophysiol. 2013; 1554–1566. 10.1152/jn.00675.2012 23843433

[21] Weber AI, Saal HP, Lieber JD, Cheng J-W, Manfredi LR, Dammann JF, et al Spatial and temporal codes mediate the tactile perception of natural textures. Proc Natl Acad Sci. 2013;110: 17107–12. 10.1073/pnas.1305509110 24082087 PMC3800989

[22] Morley JW, Goodwin AW, Darian-Smith I. Tactile discrimination of gratings. Exp Brain Res. 1983;49: 291–299. 10.1007/bf00238588 6832261

[23] Smith AM, Chapman CE, Deslandes M, Langlais J-S, Thibodeau M-P. Role of friction and tangential force variation in the subjective scaling of tactile roughness. Exp brain Res. 2002;144: 211–23. 10.1007/s00221-002-1015-y 12012159

[24] Callier T, Saal HP, Davis-Berg EC, Bensmaia SJ. Kinematics of unconstrained tactile texture exploration. J Neurophysiol. 2015;113: 3013–3020. 10.1152/jn.00703.2014 25744883 PMC4416617

[25] Pei Y-C, Bensmaia SJ. The neural basis of tactile motion perception. J Neurophysiol. 2014 10.1152/jn.00391.2014 25253479 PMC4269710

[26] Dallmann CJ, Ernst MO, Moscatelli A. The role of vibration in tactile speed perception. J Neurophysiol. 2015;114 10.1152/jn.00621.2015 26424580 PMC4686298

[27] Lieber JD, Bensmaia SJ. High-dimensional representation of texture in somatosensory cortex of primates. Proc Natl Acad Sci. 2019 10.1073/pnas.1818501116 30718436 PMC6386651

[28] Boundy-Singer ZM, Saal HP, Bensmaia SJ. Speed Invariance of Tactile Texture Perception. J Neurophysiol. 2017;118 10.1152/jn.00161.2017 28724777 PMC5646196

[29] Delhaye BP, Long KH, Bensmaia SJ. Neural Basis of Touch and Proprioception in Primate Cortex. Comprehensive Physiology. 2018;8:1575–1602. 10.1002/cphy.c170033 30215864 PMC6330897

[30] Stone LS, Thompson P. Human speed perception is contrast dependent. Vision Res. 1992;32: 1535–1549. 10.1016/0042-6989(92)90209-2 1455726

[31] Smith AT, Edgar GK. The influence of spatial frequency on perceived temporal frequency and perceived speed. Vision Res. 1990;30: 1467–1474. 10.1016/0042-6989(90)90027-i 2247956

[32] Diener HC, Wist ER, Dichgans J, Brandt T. The spatial frequency effect on perceived velocity. Vision Res. 1976;16: 169–176. 10.1016/0042-6989(76)90094-8 1266057

[33] Campbell FW, Maffei L. The influence of spatial frequency and contrast on the perception of moving patterns. Vision Res. 1981;21: 713–721. 10.1016/0042-6989(81)90080-8 7293002

[34] Bensmaia SJ, Killebrew JH, Craig JC. Influence of visual motion on tactile motion perception. J Neurophysiol. 2006;96: 1625–37. 10.1152/jn.00192.2006 16723415 PMC1839045

[35] Priebe NJ. Tuning for Spatiotemporal Frequency and Speed in Directionally Selective Neurons of Macaque Striate Cortex. J Neurosci. 2006;26: 2941–2950. 10.1523/JNEUROSCI.3936-05.2006 16540571 PMC2532672

[36] Livingstone MS, Conway BR. Contrast affects speed tuning, space-time slant, and receptive-field organization of simple cells in macaque V1. J Neurophysiol. 2007;97: 849–857. 10.1152/jn.00762.2006 17108092 PMC2636564

[37] Cleland BG, Lee BB. A comparison of visual responses of cat lateral geniculate nucleus neurones with those of ganglion cells afferent to them. J Physiol. 1985;369: 249–268. 10.1113/jphysiol.1985.sp015899 4093882 PMC1192647

[38] Mikami A, Newsome WT, Wurtz RH. Motion selectivity in macaque visual cortex. I. Mechanisms of direction and speed selectivity in extrastriate area MT. J Neurophysiol. 1986;55: 1308–27. 10.1152/jn.1986.55.6.1308 3016210

[39] Priebe NJ, Cassanello CR, Lisberger SG. The Neural Representation of Speed in Macaque Area MT/V5. J Neurosci. 2003;23: 5650–5661. 10.1523/JNEUROSCI.23-13-05650.2003 12843268 PMC2553808

[40] Zanker JM, Srinivasan MV, Egelhaaf M. Speed tuning in elementary motion detectors of the correlation type. Biol Cybern. 1999;80: 109–16. 10.1007/s004220050509 12440388

[41] Dror RO, O’Carroll DC, Laughlin SB. Accuracy of velocity estimation by Reichardt correlators. J Opt Soc Am A Opt Image Sci Vis. 2001;18: 241–252. 10.1364/JOSAA.18.000241 11205969

[42] Borst A, Haag J, Reiff DF. Fly motion vision. Annu Rev Neurosci. 2010;33: 49–70. 10.1146/annurev-neuro-060909-153155 20225934

[43] Liu J, Newsome WT. Correlation between Speed Perception and Neural Activity in the Middle Temporal Visual Area. J Neurosci. 2005;25: 711–722. 10.1523/JNEUROSCI.4034-04.2005 15659609 PMC6725331

[44] Costanzo RM, Gardner EP. A quantitative analysis of responses of direction-sensitive neurons in somatosensory cortex of awake monkeys. J Neurophysiol. 1980;43: 1319–1341. 10.1152/jn.1980.43.5.1319 6768849

[45] Ruiz S, Crespo P, Romo R. Representation of moving tactile stimuli in the somatic sensory cortex of awake monkeys. J Neurophysiol. 1995;73: 525–37. 10.1152/jn.1995.73.2.525 7760116

[46] McIntyre S, Birznieks I, Vickery RM, Holcombe AO, Seizova-Cajic T. The tactile motion aftereffect suggests an intensive code for speed in neurons sensitive to both speed and direction of motion. J Neurophysiol. 2016;115: 1703–1712. 10.1152/jn.00460.2015 26823511 PMC4808137

[47] Delhaye BP, Hayward V, Lefèvre P, Thonnard J-L. Texture-induced vibrations in the forearm during tactile exploration. Front Behav Neurosci. 2012;6: 37 10.3389/fnbeh.2012.00037 22783177 PMC3390558

[48] Manfredi LR, Baker AT, Elias DO, Dammann JF, Zielinski MC, Polashock VS, et al The effect of surface wave propagation on neural responses to vibration in primate glabrous skin. PLoS ONE. 2012;7: e31203 10.1371/journal.pone.0031203 22348055 PMC3278420

[49] Manfredi LR, Saal HP, Brown KJ, Zielinski MC, Dammann JF, Polashock VS, et al Natural scenes in tactile texture. J Neurophysiol. 2014;111: 1792–1802. 10.1152/jn.00680.2013 24523522

[50] Lieber JD, Xia X, Weber AI, Bensmaia SJ. The neural code for tactile roughness in the somatosensory nerves. J Neurophysiol. 2017;118: 3107–3117. 10.1152/jn.00374.2017 28855289 PMC6148298

[51] Lederman SJ. Auditory texture perception. Perception. 1979;8: 93–103. 10.1068/p080093 432084

[52] Talbot WH, Darian-Smith I, Kornhuber HH, Mountcastle VB. The sense of flutter-vibration: comparison of the human capacity with response patterns of mechanoreceptive afferents from the monkey hand. J Neurophysiol. 1968;31: 301–34. 10.1152/jn.1968.31.2.301 4972033

[53] Muniak MA, Ray S, Hsiao SS, Dammann JFJF, Bensmaia SJ. The neural coding of stimulus intensity: linking the population response of mechanoreceptive afferents with psychophysical behavior. J Neurosci. 2007;27: 11687–99. 10.1523/JNEUROSCI.1486-07.2007 17959811 PMC6673240

[54] Harvey MA, Saal HP, Dammann JF, Bensmaia SJ. Multiplexing Stimulus Information through Rate and Temporal Codes in Primate Somatosensory Cortex. PLoS Biol. 2013;11: e1001558 10.1371/journal.pbio.1001558 23667327 PMC3646728

[55] Saal HP, Delhaye BP, Rayhaun BC, Bensmaia SJ. Simulating tactile signals from the whole hand with millisecond precision. Proc Natl Acad Sci U S A. 2017;114: E5693–E5702. 10.1073/pnas.1704856114 28652360 PMC5514748

[56] Delhaye BP, Xia X, Bensmaia SJ. Rapid geometric feature signaling in the simulated spiking activity of a complete population of tactile nerve fibers. J Neurophysiol. 2019 10.1152/jn.00002.2019 30943102 PMC6620699

[57] Victor JD, Purpura KP. Metric-space analysis of spike trains: theory, algorithms and application. Netw Comput Neural Syst. 1997;8: 127–164. 10.1088/0954-898X_8_2_003

[58] Johansson RS, Vallbo AB. Tactile sensibility in the human hand: relative and absolute densities of four types of mechanoreceptive units in glabrous skin. J Physiol. 1979;286: 283–300. 439026 10.1113/jphysiol.1979.sp012619 PMC1281571

[59] König P, Engel AK, Singer W. Integrator or coincidence detector? The role of the cortical neuron revisited. Trends Neurosci. 1996;19: 130–137. 10.1016/S0166-2236(96)80019-1 8658595

