# Supplementary material for: Feeling fooled: Texture contaminates the neural code for tactile speed
Source: PLoS Biol. 2019 Aug 27;17(8):e3000431. doi: 10.1371/journal.pbio.3000431 (PMC6711498; doi:10.1371/journal.pbio.3000431)
Supplement: S1 Table — (DOCX) [file pbio.3000431.s008.docx]

| Textures | Exp 1a | Exp 1b | Exp 2a | Exp 2b | Nerve | Cortex | Vibrometry |
| --- | --- | --- | --- | --- | --- | --- | --- |
| Hucktowel | 1 | 1 |  |  | 1 | 1 | 1 |
| Wool Blend | 1 |  | 1 | 1 | 1 |  | 1 |
| Corduroy (Thick Ridges) | 1 |  | 1 | 1 | 1 |  | 1 |
| Chiffon | 1 | 1 |  |  | 1 | 1 | 1 |
| Nylon | 1 | 1 |  |  | 1 | 1 | 1 |
| Vinyl | 1 |  | 1 | 1 | 1 |  | 1 |
| Microsuede | 1 |  | 1 | 1 | 1 |  | 1 |
| Stretch Denim | 1 | 1 | 1 | 1 | 1 | 1 | 1 |
| Corduroy (Thin) |  |  | 1 | 1 | 1 |  | 1 |
| Metallic Silk | 1 |  | 1 | 1 | 1 |  | 1 |
| Fuzzy Upholstery |  | 1 |  |  | 1 | 1 | 1 |
| City Lights | 1 | 1 |  |  | 1 | 1 | 1 |
| Deck Chair |  | 1 |  |  |  | 1 |  |
| Dots / 1 Grating |  | 1 |  |  |  | 1 |  |
| Dots / Blank |  | 1 |  |  |  | 1 |  |
| Faux Croc Skin |  | 1 |  |  |  | 1 |  |

S1 Table. List of textures and experiments
